# LIFE-SPACE MOBILITY AS A PREDICTOR OF MEDICARE UTILIZATION AMONG COMMUNITY-DWELLING OLDER MEXICAN AMERICANS

**DOI:** 10.1093/geroni/igz038.2920

**Published:** 2019-11-08

**Authors:** Jason P Johnson, Lin-Na Chou, Yong-Fang Kuo, Kenneth Ottenbacher, Soham Al Snih

**Affiliations:** 1 University of Texas Medical Branch, Galveston, Texas, United States; 2 University of Texas Medical Branch at Galveston, Galveston, Texas, United States

## Abstract

Hispanics are a large and growing group of older adults, with higher rates of morbidity and disability than other racial/ethnic groups. Mexican Americans make up more than half of this population and are well represented in the Hispanic Established Populations for the Epidemiologic Study of the Elderly (HEPESE) survey, a longitudinal study of community-dwelling older Mexican Americans. The University of Alabama Birmingham Life-Space Assessment (LSA) is a measure assessing patterns of functional mobility in and around the home, neighborhood, and community. This study addresses the gap in research of life-space mobility and healthcare utilization with linked insurance claims data. Four hundred eight participants with 1-year continuous Medicare enrollment from wave 7 (2010) of the HEPESE were linked with Medicare claims. Logistic regression analysis was used to estimate the odds ratio of hospitalization and ER admissions. Negative binomial regression was used to estimate the rate ratio of physician visits. LSA score ranges 0 to 120, with higher scores indicating greater life-space mobility; LSA was analyzed as a 10-point decrease or dichotomously as restricted ≤59 or not restricted ≥60. A restricted LSA score among older Mexican American Medicare beneficiaries was associated with OR of 2.73 for hospitalizations (95% CI= 1.18-6.31). In addition, a 5-point decline in LSA score was associated with OR of 1.12 for hospitalizations (95% CI= 1.04-1.22). LSA score was not significantly associated with ER admission or physician visit. Interventions aimed to increase mobility in the home and the community may reduce the risk of hospitalizations in this population.

